# Sports participation volume and psychosocial outcomes among healthy high school athletes

**Published:** 2020-08-05

**Authors:** Alexia G. Gagliardi, Gregory A. Walker, Katherine S. Dahab, Corrine N. Seehusen, Aaron J. Provance, Jay C. Albright, David R. Howell

**Affiliations:** Sports Medicine Center, Children’s Hospital Colorado, Aurora, CO, USA

**Keywords:** adolescence, quality of life, depressive symptoms, athletics, youth sports

## Abstract

**Objectives:**

We assessed the association between hours/week of sports participation and psychosocial outcomes among high school athletes. We hypothesized that more hours of participation would be associated with the lower levels of anxiety and depressive symptoms.

**Methods:**

Participants completed the Patient-reported Outcomes Measurement Information System (PROMIS) Pediatric Profile 25 quality of life and other questionnaires to assess sports participation, socioeconomic status, and health history. We evaluated the multivariable relationship between hours/week in sport and PROMIS scores while adjusting for the independent effect of age and varsity team status.

**Results:**

A total of 230 high school athletes participated in this study (mean=15.4±1.2 years of age). More hours/week playing sports were significantly associated with the lower levels of depressive symptoms (coefficient=−0.073, 95% CI=−0.137, −0.010; P=0.02). Sports participation was not significantly associated with any other psychosocial domain scores on the PROMIS questionnaire.

**Conclusion:**

More hours of sports participation were significantly associated with the lower depressive symptoms, but no other psychosocial domain. While our findings are cross-sectional, sport participation may play a role in attenuating symptoms of depression in high school athletes.

**Relevance for patients:**

Sports participation may play a beneficial role in lessening depressive symptoms among healthy high school students.

## 1. Introduction

Regular physical activity has been reported to improve physical and mental health outcomes among adolescents (ages 10-19 years old) [1-5]. Exercise interventions aimed to affect psychosocial outcomes appear to reduce depression and anxiety symptoms among children and adolescents [6,7]. Likewise, frequent physical activity has been associated with improved resilience from depressive symptom development in this age group [8,9].

Previously, researchers have investigated the role of sport-specific physical activity among adolescent populations. Participation in sport has been consistently associated with numerous mental health benefits among children and teens, including increased self-esteem, social connectedness, psychological resilience, and decreased depression [10-25]. The notion that aspects of social support in youth sports may contribute to positive mental health impacts remains a likely consideration [1,9,23,24]. As youth sports participation increases, further knowledge regarding how athletics may help to improve the mental health of younger generations may allow administrators, coaches, and parents to optimize youth sport programming.

Among teenagers, 20% report a lifetime prevalence of depression by the end of adolescence, and up to 25% suffer from anxiety [24,26]. Thus, understanding modifiable protective factors against depression and anxiety in this age group are valuable. Although the previous observations suggest sport participation may mitigate negative mental health symptoms in adolescents, further study is required to understand the generalizability of these findings and the various affected aspects of mental and physical health. Therefore, the objective of our cross-sectional study was to assess the association between the number of hours per week a high school athlete reports playing their primary sport in-season and perceptions of self-reported psychosocial and physical outcomes based on the week before the assessment including physical function mobility, anxiety, depressive symptoms, fatigue, peer relationships, and pain interference. We hypothesized that more time spent participating in sport would be associated with fewer adverse health symptoms in each of the aforementioned psychosocial and physical domains assessed on the Patient-reported Outcomes Measurement Information System (PROMIS) Pediatric Profile 25 quality of life (QoL) questionnaire.

## 2. Methods

### 2.1. Study design and participants

After the Institutional Review Board approval, we approached high school athletes for enrollment in our study during their annual pre-season physical examination (PPE) day. Students were from multiple schools within a single school district of the greater Denver-metro area, between the ages of 13 and 18 years, and received full clearance to participate in sports at the time of examination. Potential participants were excluded for a history of neurological disorders, seizure disorders, or current symptoms resulting from a recent concussion. Participants were not excluded on the basis of past or current mental health conditions. Informed consent and assent for participants under 18 years of age were obtained before completion of any study documents.

### 2.2. Instrumentation and procedures

Enrolled study participants completed questionnaires with pen on paper in an open gymnasium during the PPE. Among the standardized questionnaires, students completed a health history form to assess their primary sport and the number of hours per week spent participating in their primary sport in-season, as well as their level of competition, gender, age, grade, body mass index, and medical history. For our study, “in season” refers to the time period, in which the athlete practices and competes with their respective high school sport team. Participants also completed a sports history questionnaire that asked them to report both current and past sports played, as well as when they were played. Study participants also completed the PROMIS Pediatric Profile 25 QoL [27,28]. This questionnaire has been validated for pediatric and adolescent populations and previously used specifically to study psychosocial outcomes in young athletes [29,30]. All questionnaires were self-reported with exception to the height and weight, which were recorded by an athletic trainer or physical therapist on-site during the PPE day. Parents and research staff were available to assist participants to answer any questions that the participant did not understand.

Athletes reported the number of hours per week that they expect to participate in their primary sport in-season. The health history form assessed this variable by asking, “During the season of the “primary sport” listed above, indicate the total number of hours the athlete spends training and competing per week.” If a range of hours was recorded, the average of the range was included in our database (i.e., “12-13 h” was recorded as “12.5 h”). Of note, this self-reported expected time commitment was recorded in May near the end of the school year, and athletes were not necessarily currently participating in their primary sport. As well, new/younger athletes were asked to report their expected participation before the start of their first high school season.

We assessed the number of reported symptoms of several different psychosocial domains using the PROMIS Pediatric Profile 25 QoL as our main outcome variable [27,28]. The PROMIS Pediatric Profile 25 QoL assesses six domains: Physical function mobility, anxiety, depressive symptoms, fatigue, peer relationships, and pain interference and includes a pain scale that ranges from zero (no pain) to ten (worst pain imaginable). Each question has five response options, ranging from 0 to 4, and each of the aforementioned domains includes four questions (i.e., zero equals “with no trouble” or “never” and four equals “not able to do” or “almost always”). The total raw score within each domain is calculated as the sum of all responses that range from 0 to 24, where a higher score represents an increased experience of the domain being measured. Each domain included on the PROMIS Pediatric Profile 25 QoL, with exception to pain interference, is validated and reliable (marginal reliability = 0.88) in the pen and paper format for individuals 8-17 years of age [27,28,31].

Potential covariates considered in our analysis were age, gender, body mass index, level of competition (varsity/non-varsity), history of previous injuries, and team versus individual sport, the total number of sports each participant has played during their lifetime and socioeconomic status. Participants reported age, gender, and level of competition (varsity vs. non-varsity) on the health history form.

We also assessed a history of previous sport-related orthopedic injuries and concussions. Participants self-reported “yes” or “no” answers to the following questions: (1) “Have you ever had an injury to a bone, muscle, ligament, or tendon that caused you to miss a practice or a game?,” (2) “Have you ever had any broken or fractured bones or dislocated joints?,” (3) “Have you ever had an injury that required x-rays, MRI, CT scan, injections, therapy, a brace, a cast, or crutches?,” and (4) “Have you ever had a stress fracture?.” Participants described their concussion history by answering, “Have you ever had a head injury or concussion?” followed by “How many concussions have you been diagnosed with?”

We assessed participation in primary sport using the health history form with a free-text question: “Primary Sport (Sport Athlete is most competitive in-please list one).” For the purpose of this analysis, a team sport was defined as a sport that typically requires three or more players on each team who compete concurrently (i.e., soccer, football, and baseball) [32]. An “individual sport” was defined as any sport in which only one or two athlete(s) participates for the same team during the competition (i.e., track, swimming, and tennis). We chose to include team versus individual sport in our univariable analysis because previous work demonstrates team sport involvement that may protect against depressive symptoms in adolescents, but it remains unclear if improved mental health associated with physical activity may actually be due in larger part to the social aspects of athletics [9,33]. We also assessed that the total number of sports each participant has played during their lifetime on the sports history questionnaire: “Please list the organized sports (structured time with a coach present) that you have participated in your life.”

Another factor that may affect leisure-time physical activity level is socio-economic status [34]. In our study, socio-economic status was evaluated with a validated six-item Family Affluence Scale (FAS) [35-38]. The scale assesses material assets and common indicators of wealth. Answers were assigned points, and a higher score on the FAS indicated higher socio-economic status [39].

All statistical tests were two-sided, evaluated at a significance level of a = 0.05, and performed using Stata version 15 (StataCorp, College Station, TX). Continuous variables are presented as mean (95% confidence interval and range), categorical variables are presented as the number included and corresponding percentage. We first assessed the relationship between hours per week in sport and demographic variables using linear regression models. We then assessed the univariable relationship between hours per week in sport and PROMIS score outcomes using negative binomial regression models, given the zero inflated count nature of PROMIS scores. Finally, we evaluated the multivariable relationship between hours per week in sport and PROMIS scores while adjusting for potential confounding variables. Our predictor variable was hours per week in sport, our outcome variable was each PROMIS sub-scale score, and covariates included those demographic factors that demonstrated a potentially significant univariable relationship with hours per week in sport, defined as *P*<0.20, to screen for any potentially relevant variables, rather than to test a specific hypothesis [40]. All regression analyses upheld model assumptions, as the conditional means were not equal to the conditional variances (dispersion parameter held constant). To assess collinearity, we evaluated condition indices and variance inflation factors (VIF). A condition index >30 was determined to require individual collinearity assessments, which were performed using VIF.

## 3. Results

**Figure 1:**
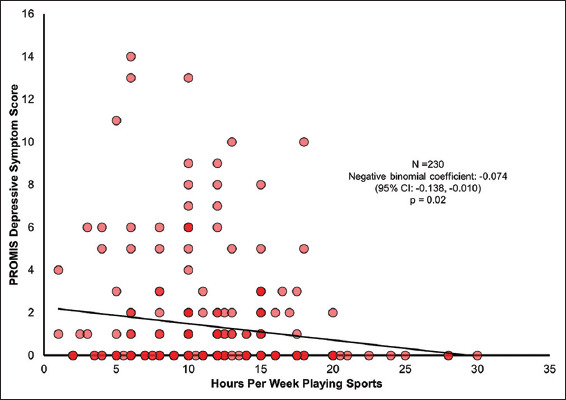
Scatterplot describing the univariable relationship between hours per week playing sports and depressive symptoms.

A total of 230 high school athletes completed the assessment (Table 1). Older age and varsity participation were significantly associated with more time spent playing sports (Table 1). The number of hours per week spent playing a sport in-season was not associated with the number of lifetime sports played; FAS scale score, gender, history of time-loss orthopedic injury, history of concussion, or type of sport played (Table 1). The univariable analysis showed a significant association between more hours/week playing sports and lower levels of depressive symptoms (Figure 1 and Table 2). Hours/week playing sports was not significantly associated with any other PROMIS domain scores (Table 2). After adjusting for the independent effect of age and varsity team status, more hours/week playing sports was significantly associated with the lower levels of depressive symptoms (Table 3). As such, our results suggest that for a one-unit increase in PROMIS depressive symptom score, the expected log count of the number of hours/week in sport decreases by 0.073 **(**95% CI: −0.137, −0.010**)**. However, hours/week plays sports were not significantly associated with physical function/mobility, anxiety, fatigue, peer relationship, pain interference, or pain intensity (Table 3).

**Table 1:** Demographic factors associated with hours per week playing sports. P values represent the univariable association between the factor listed and number of hours per week playing sports.

Continuous variables	Mean (95% CI)	Range	Association with hours per week playing sports (linear regression)		

			Negative binomial coefficient ^one degree of freedom^	95% CI	*P* value
Time playing sports (hours per week)	11.6 (11.0, 12.3)	1-30	-	-	-
Age (years)	15.4 (15.3, 15.6)	12.5-18.1	0.76	0.22, 1.29	0.006*
Number of lifetime sports played	3.1 (2.9, 3.3)	1-8	0.08	-0.38, 0.55	0.72
Family affluence scale score	9.7 (9.33, 9.85)	3-13	0.13	-0.57, 0.29	0.53

**Categorical variables**	***n***	**%**	**Negative binomial coefficient ^one degree of freedom^**	**95% CI**	***P* value**

Female gender	102	44	−0.08	−1.37, 1.21	0.90
Varsity athlete	85	37	1.95	0.64, 3.25	0.004*
History of time-loss orthopedic injury	102	44	0.26	−1.03, 1.55	0.69
History of concussion	52	23	0.11	−1.43, 1.65	0.89
Team sport athlete	162	70.1	0.82	−0.58, 2.22	0.25

* *P*<0.20 and included as a covariate in negative binomial regression models

**Table 2:** Univariable associations between hours per week playing sports and PROMIS domain scores (negative binomial regression models).

PROMIS Domain	Median [IQR]	Negative binomial coefficient ^one degree of freedom^	Standard error	95% CI	*P* value
Physical function/mobility	0 [0, 0]	0.028	0.043	−0.057, 0.113	0.52
Anxiety	1 [0, 4]	−0.025	0.019	−0.063, 0.013	0.20
Depressive symptoms	0 [0, 2]	−0.074	0.033	−0.138, −0.010	0.02*
Fatigue	1 [0. 3]	−0.030	0.022	−0.072, 0.012	0.16
Peer relationships	15 [12, 16]	0.000	0.004	−0.008, 0.008	0.96
Pain interference	0 [0, 2]	0.014	0.033	−0.079, 0.051	0.67
Pain intensity	0 [0, 2]	−0.002	0.027	−0.054, 0.051	0.95

**Table 3:** Multivariable associations between hours per week playing sports and PROMIS domain scores after adjusting for the effect of age and competition level (negative binomial regression models).

PROMIS Domain	Negative binomial coefficient^three degrees of freedom^	Standard error	95% CI	*P* value
Physical function/mobility	0.027	0.045	−0.061, 0.115	0.55
Anxiety	−0.028	0.020	−0.664, 0.011	0.16
Depressive symptoms	−0.073	0.033	−0.137, -0.010	0.02*
Fatigue	0.034	0.022	−0.077, 0.008	0.11
Peer relationships	0.001	0.004	−0.007, 0.009	0.84
Pain interference	−0.006	0.034	−0.072, 0.060	0.86
Pain intensity	0.006	0.027	−0.047, 0.059	0.83

* Lower depressive symptoms were significantly associated with more hours per week playing sports (*P*<0.05)

## 4. Discussion

Among our cohort of high school athletes (ages 12.5-18.1 years old), more hours per week of in-season participation in their primary sport was significantly associated with lower self-reported depressive symptom ratings, even after accounting for age and competition level. However, hours of participation and physical function mobility, anxiety, fatigue, peer relationships, or pain interference were not associated. Despite statistical significance, it is difficult to assess clinical significance in this finding. Although statistically significant, the association was weak. More robust research is required to reliably assess the clinical impact of increased sports participation volume. In part, our results align with previous research demonstrating the psychological benefits associated with sports participation in an adolescent age group [9-22,24,25]. Although the cross-sectional design of our study does not permit causal interpretation of our findings, our findings support the notion of past work that sports participation may be one of many factors associated with fewer depressive symptoms. Considering that level of competition and type of sport (team vs. individual) were not associated with symptoms of depression in our cohort, increasing access for students to participate in any level or type of organized athletics may improve, or at the least not produce harmful effects, related to mental health. A balance is required; however, as recent work suggests that higher volumes of sport participation may also lead to a higher rate of sport-related injury [41,42].

In contrast to the previous research, our results do not suggest higher volumes of sports participation that will universally be associated with all aspects of physical and mental health – specifically physical function, anxiety, fatigue, peer relationships, and pain interference [24,25,43,44]. This discrepancy may be partly due to the PROMIS instrument, which assesses state characteristics (e.g., within the past 7 days), to identify symptoms in our sample. Further, athletes completed the PROMIS in May, not necessarily during their season. As the PROMIS reflects the past 7 days only, the questionnaire may not have captured in-season effects of sports participation on depressive symptoms. Survey fatigue may also figure into our results, as students may not have carefully considered their rating for each question. We also found no difference in psychosocial outcomes between team sport and individual sport athletes. The previous research suggests improved mental health associated with team sports compared to individual sports [24,45-47]. This inconsistency between our results and previous work leads us to consider the possibility that the previous findings may not be generalizable to our cohort due to unique aspects of our cohort’s community and extracurricular activities outside of athletics. These environmental factors, possibly including socioeconomic status, weather, and geography unique to the greater Denver-metro area, may mediate the psychological and physical domains measured by the PROMIS questionnaire.

Within our univariable analysis, we determined non-significant and weak associations between the number of hours per week playing a sport and lifetime sports participation, FAS score, gender, time loss from sport due to orthopedic injury, or concussion. This suggests that sports participation time commitment may not be determined by the previous athletic involvement or other aforementioned factors. This indicates that students may successfully modify their frequency and volume of participation regardless of the previous experience. It is recommended that children participate in at least 60 min of moderate to strenuous exercise daily, but high school athletes may benefit from more time in a sport-specific setting that provides positive social interaction and social development [5,45]. However, it has recently been suggested that youth athletes should not participate in more hours per week of sport than their age [48]. As such, community and school programming will play an important role in enabling adequate sports participation among adolescents.

There are several limitations to our study. First, the cross-sectional design prevents any predictive or causal interpretations. Second, we did not collect information on the participant’s activity level near the time point of the PPE day and cannot assess the possible association between current activity level and outcome measures. Third, the PROMIS questionnaire is self-reported in a public gymnasium, and the lack of privacy may have contributed to self-report inaccuracies. The PROMIS also reflects the current state of the athlete (within the past 7 days) and may be susceptible to variability based on life events occurring in the proximity of the assessment. Fourth, as our study was conducted among high school athletes at a single school district, the generalizability of our findings may not be applicable to other geographically or demographically diverse populations. In addition, the questionnaires were administered in May near the end of the school year, and thus, data were susceptible to recall error for athletes not currently participating in their primary sport and young or new athletes may provide a less accurate estimation of their likely participation volume for the upcoming season. Another limitation of our study is that some athletes reported a range of hours, for which the average was recorded in the database rather than the exact number of participation hours. Furthermore, we did not ask about the length of time participants that were engaged in various sports or physical education or activity classes, leading to potential variability in the magnitude of this factor. Finally, participants were not screened for the current or previous mental health conditions, which may confound our outcome variables.

## 5. Conclusion

More hours spent playing sports were weakly, yet significantly, associated with lower ratings of depressive symptoms within the 7 days preceding assessment, after adjusting for the effects of age and competition level. Future research will help administrators, coaches, and parents further understand how to optimize youth athletics programming to benefit adolescents. While our findings are cross-sectional, they may be useful to providers, educators, or coaches involved with high school athletes in working to optimize youth athletics programming. It is likely the psychological benefits commonly found to be associated with sport participation that is partly due to the social aspects of youth athletics, and thus, adequate sport participation may improve psychosocial outcomes among high school students regardless of the sport and should be encouraged and made accessible to all adolescents [1].
